# Mechanical Dentistry

**Published:** 1875-10

**Authors:** O. Willson

**Affiliations:** Aurora, Ill.


### ARTICLE II.

### *Mechanical Dentistry.*

BY O. WILLSON, AURORA, ILL.

Read before the Illinois State Dental Society.

In offering for your considerations some suggestions relative to Mechanical Dentistry, the fact seems to gather new force that it is quite possible for us, as a profession, to carry with our services a too sanguine idea of permanence. It may be we do not talk sufficiently to our patients. The tooth filled with metal is not only as good as a sound tooth in the same mouth, which is the more reasonable expectation, but the repaired surface is *insured from further destruction.* Whereas, a close canvass of the case in point would, like the risks of express and insurance companies, surround it with several "conditions."

The plate of artificial teeth adapted after the lapse of eight or ten months from removal of the roots is termed *permanent*, and so there comes about a belief that the work is good for all time—hence the idea of warranting, practiced by the more unskillful or unscrupulous.

The "temporary plate," is so named from diminishing size of alveolar border, immediately after extraction of the teeth—how few there are of any other than temporary plates. The breaking-down of process, of course is most rapid during the first weeks subsequent to extraction, but it is, especially under non-conducting plates, the beginning generally of a *ceaseless* reduction. Pertinent, is the recent inquiry of one of this Society. "What may our patients reasonably expect of us?"

Of late years, particularly since the introduction of rubber as a base for artificial dentines, mechanical and operative dentistry have exhibited a growing tendency to separate. This has been eminently noticed. Dr. Eames, you recollect says in the March number of the *Missouri Dental Journal*, for 1874: "The maker of artificial teeth will one day occupy the same relation to the dentist that the manufacturer of wooden legs does to the surgeon." This remark, you notice is prefaced with a truthful picture of the present gloomy status of mechanical dentistry, and that the prospective operator or laboratory-workman should have, if desired, a separate course of study in college and different degree. Very many of our profession entertain a similar opinion.

This disintegration tendency has probably escaped the notice of but few of you; the cause may be less apparent, and given a *remedy*, some shall *yet* counsel *divorce*,—but whether it is to be in school, or office, or in both; the interest of all demands an earnest inquiry into the elements of this "low ebb" in mechanical dentistry, that, if possible it may be remedied. Prominent among these is: too great expectations of the cheap bases as yet offered.

These "expectations," on the part of the community have, upon our own as upon other professions, a preponderating influence; and are, if we mistake not—(1) for the best of professional services; (2) at a compensation within the the means of the greater number.

The exceptions to this general proposition are mainly but a difference in degree of reflection upon the subject, for the community being but the archetype of the individual, we need but ask ourselves our own desire in this direction; and the sum-total of its good judgment is commonly not far to the rear of the real merit of our performances. Measured by this standard, how far have these expectations been realized? Let us pass in rapid review the bases and teeth now in use with reference to their merits and defects.

Of the metals (if we except platina with the porcelain combination—continuous gum work) gold is the best, for either partial or full sets; for the former, it is without a peer. By reason of being readily tarnished, silver plates are largely in disfavor. Aluminum is practicable only for full sets, and will not resist the corrosive agents of some mouths. Other metals, as Ingersoll's American platinum, Wetson's metal, cheoplasty, block-tin, &c., are used with varying success. For full sets, the celluloid combination with these metals, using plain teeth, commonly gives the best satisfaction possible to them, and if the whole plate be made of celluloid or rubber, this use of plain instead of gum teeth, gives stronger work, since the material on the outer part of the *circle* is continuous; but using gum teeth divides this into segments. A substitute for the celluloid combination with metal plates is, except on silver, the English pink rubber, bleached; but since it is wanting in requisite strength, the less is used the better, confining it to parts in sight.

In cases of great prominence of upper jaw, plain teeth adjusted directly to the membrane, with a slight rim about the tips of teeth to strenghen this frail attachment, gives a very natural appearance sometimes. By keeping pledgets of cotton in the alveoli of the front of the mouth, to keep them open a few days, plain teeth may pass the plate into these holes.

But, inasmuch as neither the celluloid nor pink-rubber is translucent, together with the incidental fact that this color even cannot be relied on for permanence, their appearance is very objectionable. If gum teeth be used, and in sections of three and two, as you well know with the desired position of one, all must go; and the fewer the teeth in sections, the more of those uninviting seams between them.

While speaking upon the demerits of teeth coming *complete*, in make, to the hands of the dentist, although this is claimed, by some, to belong to the work of the operator, allow a word upon pivot teeth. But for the one fear of alveolar abscess following the insertion of pivot teeth, there would be nothing comparable with the plate-tooth gold-pivot and filling attachment for substituting the ten front teeth; and the "coming dentist" is he who can so renovate and prepare nerve canals for this purpose as to insure us comparative immunity from this threatening ill, so that we may more confidently undertake this most meritorious work.

Almost any of these metals are preferable to celluloid, rubber, gutta-percha and the like, for one important reason —they are conductors. A substance may promptly respond to thermal changes, and not be a conductor of electricity. It may not conduct either heat (and cold,) or electricity, and yet permit electrical induction;—indeed, there is no substance with which we are acquainted that can prevent or intercept its influence. These last named substances are non-conductors of both, and thus preventing the thermal changes to which the membrane covered by them is accustomed, they prevent vascular action; and the ignorant or henious practice, as the case may have been, that counseled patients to exchange their gold for rubber plates, has been an incalculable injury to the human face, to say nothing of the balance of the body, and is the entering wedge of this *divorce*. A *genuine* artist will not long cleave to the brush, paint and canvass, that belie his talent.

Nor can the adept at laboratory work be long contented with the exclusive use of materials of like tendencies,—nothing short of financial anxiety can confine him to it,—hence this branch of dentistry is, for the most part, conducted by another class, the manipulation of these bases, requiring but a medium of skill, and permitting the exercise of about the same amount of artistic taste. These non-conducting bases have *one* virtue, they are cheap and in this respect have held undisputed sway until now ; and, catering to this axiomatic duty of all, to consult their purse, we have passed the limit of wise expenditure, and mechanical dentistry is to-day paying the penalty.

A lamentable result of the use of rubber is, that under it the alveolar process recedes so rapidly, as to make frequent demands for a better adaptation of the plate to the jaw, and thus the patient continually feels the necessity of the low price and "*quick returns.*" The business is *reactionary* "grows by what it feeds upon." Nor are we to look to celluloid for *deliverance* from this untoward condition. It is stronger than rubber, and for most cases has better color ; it hugs teeth closer, and upon it *is no royalty.* Otherwise, it is open to the same objections as rubber, unless time may demonstrate its freedom from poisonous effects ; and neither celluloid nor rubber will endure the verdict of an enlightened practice, but will continue the work of depression and disintegration.

A destructible material, used simply in combination with a base susceptible to changes of temperature, is bad enough but it is much worse to form the whole of the plate of it, particularly if its ingredients are detrimental to the health of the wearer. Few have not noticed the gradual waste of rubber plates, along the labial and buccal border, in some mouths,—the exceeding tenacity of oral debris for this base, over that of metal, or porcelain, the odor of which is as intolerable as that from behind teeth soddered to the plate, golden rims, or that from deep-seated dental caries. If any be still incredulous, remove teeth from rubber plates and notice the odor behind them.

In accordance with established laws of the animal economy, immediately subsequent to extraction, the arterial supply to the capillaries is more abundant, which commence the deposit of plasma at the bottom of the alveolus, while the alveolar process above this terminal deposit is carried off by the absorbents, after the active inflammatory stage, it having served the purpose for which it was designed. The point at which this broken-down process, and the blastematic deposit meet, or the natural equilibrium of waste and supply is restored, is at the usual location of muscular attachment, but by reason of constitutional disturbance of this equilibrium,—the non-conducting and poisonous property of plates, by separation of particles, pressure, or all combined—the alveolar ridge, especially in scrofulous habits diminishes in size, as you doubtless have noticed, until where there was a ridge on the under jaw, there is practically a groove, and upon the superior maxillary the palatal bones are as prominent as the border, particularly if deep air-chambers, so-called, have been made in the plates. This reduction, *morbid* of course, it will be remembered, is effected either by insufficient capillary nutrition, from defective arterial supply, of quantity or quality, while the degree of absorption is unchanged from the natural; or where the absorbent vessels are excessively *active*, the condition of deposit remaining unaltered from the state of health, this being the condition noticeable under non-conducting bases, though any plate, by continuous irritation from unsteadiness in position or otherwise, will induce it.

A metal or porcelain plate, inserted early after extraction, and well fitted to the uneven prominences, keeps them bearing nearly the same relation to each other, and thus helps to prevent movement of the plate; and the acme of excellence in the adaptation is, for all atmospheric plates, where the pressure is *least* consistent with comfort to the wearer. Pressure, gradual and persistent, as even by good fitting plates in general, is a matter of regret, perverting to some extent vascular action, and as atmosphere is excluded, over-stimulating the absorbent vessels, and so we see even under bases compatible with membranous function, more or less reduction in size. Now add to this necessary evil the unnecessary one—a base incapable of responding to thermal changes, convicted repeatedly of poisoning, and we have a low grade of chronic inflammation from irritation, chemical and mechanical, that keeps the absorbents preternaturally active; and this reduction is co-extensive with the irritation. Such a base is worse than a *bread and mtlk poultice*, long continued.

Two other styles of work deserve prominent mention—continuous gum and porcelain; either of which seem adequate to meet the expectations of comfort and cultivated art, though neither are adapted to forming partial sets, unless the teeth are contiguous to each other. Both require a cultivation of skill to manipulate them successfully, seldom met with in these degenerate days of " Josiah's" reign ; both are immeasurably superior to any other, and strange to say, neither are in general use throughout the country. So far as we are acquainted with the work of both, we give our unqualified prefercnee to porcelain. The general appearance of each in the mouth is the same; each requires nearly the same time to manufacture it, but the porcelain has the advantage of a little less cost. It is much lighter, and the adaptation to the mouth can be made perfectly satisfactory. It is stronger, because the teeth contain no pins to expand or cut the tooth in twain, and because the teeth coming in biscuit form, enables the use of body, containing less of the glass of borax, so that the heat may be run up far higher than in making continuous gum-work, and thus is made the strongest of porcelain. We *believe* that a good piece of this work cannot be fairly broken in the mouth. Continuous gum work depends upon the platinum for strength, and this must be so braced that it will not spring, which adds greatly to the weight. This work being of the *enameled* teeth, the gum and body must contain a considerable flux, which weakness the investment, and it checks, if the plate yields.

Porcelain plates, though invented about twenty-five years since, by Loomis, of Washington, D. C., seem until recently to have been wanting in strength, and until the improvements of Dr. Dunn, some ten or twelve years since, were rather undesirable. The teeth were carved from the plate, instead of being moulded from the natural ones, as now. The color of plate and teeth were the same, making the general appearance quite defective and inartistic. The manipulation throughout is now so much improved, as to be almost another method. A model is obtained in the usual way, upon which is perfectly fitted a wax plate, with length and fullness of required teeth, with simply the prints of teeth for articulation. An articulator of plaster is now made, for fitting the plate after it comes from the furnace, also a cast from this wax extending back of the wax about an inch. The model is now trimmed, allowing for shrinkage of the biscuit; then cut out the wax in the lingual surface, refill with sheet wax, of the desired thickness for plate, which by filling with plaster, makes the whole ready for setting up the teeth. The process of baking, is analagous to that of continuous gum.

Such, gentlemen, we conceive to be an unbiased synopsis of the merits and demerits of the plates and teeth now of the Dental Laboratory. If it be an intelligent statement, then it is a fact, that gold for partial plates, and porcelain or continuous gum work for full sets of teeth, are the only bases really desirable, for the reasons that they are the only ones at once cleanly, healthy, and naturally artistic,—challenging the verdict of cultivated criticism. There construction is not of the mushroom order of attainment. It *does* require *unusual* intelligence and skill to handle these styles of plate-work efficiently; *unlimiting* genius, almost. How many of our laboratories turn out this kind of work? Do we exagerate when we say that not one in 500 of all plates made during the last ten years, have been even a *semblance* of the *best of professional service* in mechanical dentistry. Why any wonder then, that this branch of our profession is a by-word and contempt, almost wanting in mechanical and artistic skill. How few talented young men now propose to enter it; and shall it continue such, or is there for it a resurrection; and if so, how shall it be? by giving it a back seat in school, or office, or shall it be by honoring it in *both*, as its importance demands? also by individual and collective effort to inform first ourselves, and then our patients, as to their best interests in this direction using arguments similar to those we employ in the operating department. It is in vain that we attempt to ignore its claims; they are parallel with those of operative dentistry. So long as teeth decay, so long will somebody fail to care for them successfully; and so long as pride and good sense endure, so long will there be a demand "in the upper stories" for good mechanical dentists; and to lower the standard of excellence in education or skill, or to encourage our best talent to shun this branch of our profession, will but enhance the evils we seek to avert.

Whoever has been in the practice of dentistry a sufficient time to give to his best efforts a fair trial, must have often passed in astonishment, not only at his many failures, but the varied and extended field of investigation suggested by it; and fortunate indeed is he whom the revelation was not so astounding as to drive into discouraged apathy.

The blending of art, surgery and mechanics in our specialty, is so intimate that the point of divergence is often difficult of discernment. Thus in addition to the ordinary work of constructing and applying artificial dentures, the mechanical dentist is often called upon to insert pivot teeth, make appliances for changing the position of natural ones, construct and apply palatal obturators, and not infrequently to devise splints for maxillary fractures, and sometimes to restore lost portions of the face; also to suggest and use means of reducing luxation, congenital and accidental. Surely, here is need of scholarly attainment, and when to the knowledge thus suggested, that of the minerals is necessarily added, we do not consent to mechanical dentistry in its best estate, as being the inferior of any calling. But if A. can only manufacture an article that B., C., or D. can, without especial effort, then is it subject to the competitions of ordinary *trades.*

Mechanical dentists should have as thorough training as operators, if indeed the course of study be not identical, which is very necessary, in case of separate and independent offices for these two branches; but where they are practiced in the same office, it is possible to be fairly successful, if the laboratory man is wanting in a knowledge of some things, that in an operator would be empericism. This, however, necessitates the latter to consult with every patient. Each *should be capable* of giving good counsel, for either plate-work, &c., or operating. Their relation is more intimate than surgery and wooden legs.

In our own office we connect the extracting, with the work of the laboratory ; and after trying this plan for eleven years, feel sure it is the better way.

Dr. Swain, in a paper presented to this Society in 1872, remarked that he who had cared for the teeth of a family or individuals, for years, is or should be, the best judge of replacing them. Who that observes, can fail to concur in this statement. But since it is more convenient to divide the work, the evident necessity is still to keep the two branches in the same office, thus uniting talent, for though "like peevish man and wife together jar—they yet are both to part."

The rejuvenation of mechanical dentistry rests in the realm of exalted skill, and a cultivated public opinion; though the *price* of that skill, must necessarily be as variable as the location of its exhibition. That which is a fair remuneration in one place, would be exorbitant in another; while yet, it may be true, that skill is not subject to the fluctuations of ordinary merchandise.

For this education of the masses, we must wait,—never forgetting that 'tis we ourselves who should and do, to a great extent, mold public opinion upon our services. Hence the necessity of thorough preparation for our work ; and as we are enabled to offer an article *in every way commendable*, at a reachable figure, so will the incompetent of the laboratory seek other avenues of employment, and mechanical dentistry be lifted by degrees, from this "slough of despond" towards which it now gravitates, up to a position of artistic taste and enduring utility, that shall rank quite favorably with her twin-sister, the operative department.

---

### SELECTED ARTICLES.

